# Participant Engagement in a Transmedia Storytelling Web-Based App Intervention for Mental Health of Latina Women: Qualitative Analysis

**DOI:** 10.2196/22575

**Published:** 2021-01-13

**Authors:** Patricia D Soderlund, Adrienne S Martinez Hollingsworth, MarySue V Heilemann

**Affiliations:** 1 School of Medicine University of Minnesota, Duluth Duluth, MN United States; 2 Department of Internal Medicine, UCLA School of Medicine National Clinician Scholars Program University of California, Los Angeles Los Angeles, CA United States; 3 School of Nursing University of California, Los Angeles Los Angeles, CA United States

**Keywords:** transmedia, Latina, mental health, mobile applications, internet, depression, anxiety, storytelling, mobile phone

## Abstract

**Background:**

Stigma, fear, and lack of knowledge regarding treatment options or where to get help create delays for Latina women in accessing needed mental health help. Story-based media interventions hold appeal for Latina women. Thus, we drew upon the Social Cognitive Theory by Bandura to create an evidence-based, transmedia storytelling web-based app for mental health called *Catalina: Confronting My Emotions* to connect Latina women to a curated set of mental health resources. Understanding how Latina women perceive various aspects of the web-based app will help design future expansions.

**Objective:**

A previously published analysis led to the development of a category on how participants related to the lead character (Catalina) in the story line of the web-based app as a real person. However, the purpose of this analysis was to gain an understanding of participants’ experiences with the extension of the dramatic story line of the web-based app beyond Catalina to a Latina nurse-therapist character named Veronica, who was featured prominently in the app’s interactive content and bonus videos.

**Methods:**

Qualitative analyses were conducted with interview data from a community-based sample of 28 English-speaking Latina women aged between 21 and 50 years who scored above the threshold for anxiety (Generalized Anxiety Disorder-7) and/or depression (Patient Health Questionnaire-9) but were not suicidal at screening. Data were collected 72 hours after participants engaged with our transmedia storytelling web-based app for mental health. Grounded theory methodology guided the analysis and interpretation of data that had been collected telephonically, recorded, and transcribed with identifiers removed. Analyses included initial and focused coding using process codes (gerund form of verbs in codes focused on action), informed by symbolic interactionism, and the development of categories with properties through constant comparison, memo writing, and the use of charts and diagrams.

**Results:**

Our participants experienced a multiphase process that was most heavily related to Veronica, the Latina nurse-therapist character in our web-based app, who led them through a process to a place of action. We conceptualized this process as moving from passive viewer to active participant of a transmedia storytelling web-based app intervention. Overall, 3 new conceptual categories provided insight into women’s experiences, including encountering a trustworthy nurse-therapist character, taking in messages that dispel old beliefs, and preparing when and how to take action. Each category has nuanced properties that reflect participants’ experiences.

**Conclusions:**

Active engagement with our web-based app led our sample to successfully transition from the viewpoint of the observer to the viewpoint of the experiencer, moving from a passive position of watching to active engagement that involved imagining, thinking, reflecting, and acting. Careful development of dramatic material for health-related web-based apps using transmedia story extension and bonus videos needs to be based on input from the target group from the start of development through evaluation and testing.

## Introduction

### Background

The median delays in seeking mental health care for depression and anxiety in the United States have been estimated to be 9 and 8 years, respectively, even if the person knows that their symptoms are due to a mental health problem; these delays were significantly associated with being Latinx or Black [1]. A meta-analysis on the duration of untreated unipolar depression showed that participants who failed to initiate care within 8 weeks of becoming aware of depression had a lower probability of responding well to treatment or achieving remission [2]. In another study, 70% of the 287 Latina women in a community-based sample delayed seeking needed physical health care; furthermore, having depression or anxiety was associated with 3.1 times greater odds of delaying needed health care (95% CI 1.6-5.9) [3]. Therefore, for Latina women, the consequences of untreated depression and anxiety threaten both physical and mental health.

The prevalence of depression is higher among Latina women than among Latino men in the United States [4,5], as it is globally [6]. Compounding the problem, Latina women have been reluctant to seek care [7] due to fear, guilt, shame, privacy concerns, lack of family support, or distrust of professionals [8]. This includes English-speaking Latinx men and women [9], who have reported higher levels of self-stigma and have more often said they would conceal a potential mental health problem compared with non-Latinx White individuals [10]. Latinx men and women are approximately half as likely as people of other racial or ethnic groups to obtain mental health care [11]. Thus, a dynamic intervention that helps this group overcome barriers to care and identify available resources is crucial.

As mental health literacy is a segue to treatment engagement [12], it is an asset that would benefit Latina women with symptoms who wish to obtain mental health help. Key features include having the ability to recognize a mental health disorder as it develops, being clear about professional and self-help treatment options, and knowing where to obtain resources and information, as well as how to help someone else who is struggling with their mental health [13]. However, the question arises of how to most effectively reach Latina women with this content. This is especially challenging with Latina women, who avoid even talking about mental health due to fear of stigmatizing labels [12]. Nonthreatening strategies are needed to increase the confidence of Latina women and encourage them to seek help and make contact with a provider. A discreet approach to introducing and delving into the topic of mental health is likely to be more acceptable to Latina women; however, for the approach to be effective in helping Latina women achieve the goal of mental health and wellness [14], it should be uncomplicated, engaging, and directly related to getting treatment.

One strategy is to use technology that most Latina women have in their pocket, purse, or desktop, that is, their smartphone, tablet, or computer. Latinx men and women report high levels of internet use both for entertainment [15,16] and for accessing health information [17]. Latinx men and women have high rates of smartphone ownership [18], which supports the idea of a mental health web-based app intervention that is accessible on smartphones or other mobile devices and can be ultimately personalized as well as scalable to maximize reach [19]. However, any endeavor to create a web-based app needs to be guided by theory and based on scientific evidence, as Torous and Roberts [20] have explained. They cautioned that only very few of the 10,000 mental health apps that were available for the public to download in 2017 were actually based on data [21]. With such a large number of available apps, it can be quite challenging for users to find the few evidence-based apps among those that were not developed based on data [20]. In addition to being evidence-based, developers need to take extra steps to ensure that their apps are user friendly, designed with user input [22,23], and respectful of users’ privacy [21,23]. Ultimately, if the app is useful for meeting users’ needs, it holds promise for raising the otherwise low rates of engagement with mental health apps [21].

When considering a target group of symptomatic Latina women, apps also need to be culturally acceptable, desirable, and enjoyable to them, so that they will be attracted to using the apps. Latinx men and women are considered a mobile-first community with some of the highest levels of smartphone engagement. For example, 90% of all Latinx consumers use their smartphones to perform video streaming. In particular, Latina women report high levels of television watching and movie going [16], spending more than 30 hours per week watching television and more than 22 hours per week using their smartphones while exploring apps, downloading and viewing videos, or surfing the net. Latina women also report using YouTube, Google+, Instagram, Snapchat, and Twitter at higher rates than non-Hispanic White women [24]. For these reasons, we created an evidence-based, storytelling mental health intervention that could link untreated Latina women to the needed care and resources via a smartphone, tablet, or computer using transmedia. Transmedia involves telling stories that unfold and extend across multiple digital platforms, including smartphones, tablets, or computers, which offer users and viewers a media experience that has the potential to go deeper as they gain different points of view related to the story world, plot, or characters through extra scenes or bonus videos [25].

### Previous Work

Fueled by input from Latina women, informed by Social Cognitive Theory by Bandura related to media, vicarious learning, and behavior change [26], and inspired by the 1970s edutainment productions by Miguel Sabido on Televisa in Mexico [27], we created a Hollywood-quality web-based app with a responsive design that can be used on smartphones, tablets, or computers. We partnered with a programmer and Latinx media and film professionals, as described elsewhere [28,29]. Our web-based app is character driven in that the momentum of the web-based app is carried forward by the characters themselves. In addition, our characters are messengers of health-related content embedded in the story and bonus videos. This means that the story line is extended by featuring characters in additional bonus videos that are psychoeducational or therapeutic. In this way, the characters present helpful information from within the story world while also enhancing the participants’ media experience. For these reasons, we developed the characters with special attention to deidentified input from Latina women struggling with depression who participated in previous research studies [30-38]. In addition, during the design phase, we received critique and input from Latina therapists and non-Latina women therapists who worked with symptomatic Latina women to maximize usefulness from a provider point of view. These sources of input influenced all scripts, directing, and acting. In theater testing, Latina women of the target demographic gave feedback useful for editing and culturally tailoring the content. Latina focus group participants named it *Catalina: Confronting My Emotions.* Our approach heeds advice voiced by Torous et al [22,23] about the importance of a collaborative approach in app development that includes patients and providers.

A mixed methods study with 2 major aims was conducted. The first was to quantitatively analyze the feasibility, acceptability, and efficacy of the web-based app intervention, and the second was to qualitatively explore the experiences and perceptions of 28 English-speaking Latina women in a community sample that engaged with the web-based app. All participants were aged between 21 and 50 years and scored above the threshold for anxiety [39] and/or depression [40] but were not found to be suicidal at screening. Interviews were conducted by phone approximately 72 hours after participants engaged with media in the web app.

Due to the design of the home webpage of our web-based app, participants were led to engage with the videos by starting with the story-based videos about a character, Catalina, portrayed by actress Sandra Parra. These videos included a 11-minute webisode (online television episode) about Catalina struggling with feelings of sadness, frustration, and worry in her daily life. This was followed by a 3-minute video log of Catalina, making a recording for her best friend in which she confides in her best friend that she is thinking of seeking therapy. Users then watch a 3-minute extra scene of Catalina coming out of a community counseling center and follow her as she continues walking down the street while leaving a voice message for her best friend about her positive experience in therapy with a character named *Veronica*. At this point, participants have not yet seen Veronica, but they hear Catalina talk about the therapy session with Veronica as something she values.

The goal of the early videos was to attract and pique the interest of participants in the dramatic story so they would be motivated to click to open the bonus and interactive videos that extended the story through Veronica. Thus, the story involved some drama, some romance, and some tense scenes depicting difficulties Catalina had involving an old boyfriend, her mother, and her emotions. Our strategy was successful, and all 28 participants clicked to open and watch all features of the web-based app [28]. They got their first glimpse of Veronica, portrayed by actress Yareli Arizmendi, in a 4-minute bonus video in which she speaks from her point of view as a Latina and a nurse who is a therapist. Veronica looks directly into the camera as she speaks to the participants. The script allows Veronica to talk about Catalina’s situation in an accessible way and to build upon it to share how common depression and anxiety are among women and to provide various points of psychoeducation. She then invites participants to click to open a sequence of interactive videos. Over the course of 5 short 1- to 2-minute interactive videos, Veronica again speaks to the audience directly, posing questions about what is holding them back from getting care [41] to help them consider their own situation, emotional needs, and desires. Finally, Veronica invites participants to click to open her blog, written from her point of view, which contains carefully selected links to hotlines, local clinic webpages, telephone numbers, and websites that provide free or low-cost resources and information about mental health [28,29].

Our team analyzed and published an analysis of qualitative data from our sample about their perceptions of the fictional lead character of the story, Catalina, whom they related to as a real person with a past, present, and future [29]. However, as a major portion of the transmedia web-based app involved Veronica, the nurse-therapist character, it was imperative for us to analyze the qualitative data from participants about Veronica. She was the character who guided participants through all other aspects of the web-based app experience, including the transmedia bonus videos, interactive features, and resource-rich blog.

### Objectives

The purpose of this qualitative analysis is to explore, describe, analyze, and interpret the perceptions of English-speaking Latina women with elevated levels of depression and/or anxiety related to the nurse-therapist character in our transmedia web-based app, named Veronica. Through this analysis, we aim to provide knowledge for consideration in the future when developing nurse characters or other health provider characters in mental health apps designed especially for Latina women and other users struggling with depression or anxiety symptoms.

## Methods

### Design

For this qualitative analysis, we used the techniques of grounded theory methodology [42,43] to analyze interview data that were collected as part of a mixed methods intervention study. As was described elsewhere [28,29], approval was obtained from the UCLA Institutional Review Board. Symbolic interactionism [44,45] informed our use of grounded theory techniques; symbolic interactionism holds that people make sense of reality through the social aspects of their daily lives and meaning emerges through social interactions. This sensitized us during analysis to focus on what was meaningful to participants about their web-based app experience, with special attention to interactions with the characters in the context of their own lives.

### Sampling and Recruitment Procedures

To attract participants, flyers were distributed at 9 community-based sites located in a metropolitan area of Southern California. Interested women called the study phone and were screened. Purposive sampling was used with the goal of recruiting English-speaking Latina women who scored above the threshold for anxiety [39] or depression but were not suicidal [40]. Those who met the inclusion criteria and gave web-based informed consent were aged 21-50 years, could speak and read English, and had access to the internet via a tablet, computer, or smartphone.

### Data Collection

Participants completed a web-based baseline survey before engaging with the web-based app. A telephone interview was conducted up to 3 days later. A semistructured interview guide was used [29], which included open-ended questions about the Latina women’s experiences with each aspect of the transmedia web-based app, their perceptions of Veronica, and attitudes about help-seeking. Being semistructured, the interview was designed to allow participants to expand on topics as they desired. As is recommended by grounded theory methodology, participants’ answers in early interviews influenced questions posed in later interviews. Thus, additional questions that emerged in early interviews about what Latina participants found pertinent were added to subsequent interviews as data collection proceeded. All telephone interviews were conducted by a nurse scientist who is also a mental health nurse practitioner (first author). After completing several steps in the study, including the audio-recorded telephone interview (lasting an average of 45 minutes), participants received a US $60 gift card (via US mail, text message, or email). All digital recordings were password protected and were stored on a server with a firewall. Audio recordings were transcribed verbatim using a professional and secure transcription service. All transcripts were deidentified, checked for accuracy, and uploaded into Atlas.ti (qualitative data analysis; Scientific Software Development GMbH) [46]. All participants who enrolled in the study completed the entire study.

### Data Analysis

We engaged in a rigorous qualitative analysis of the data using the techniques of grounded theory methodology. Initial coding of each line of data was performed using process codes (gerunds) to maximize our focus on the actions of each participant (including their thinking and self-talk) and their point of view as an individual. This reduces tendencies to project beyond the data or to make an interpretive leap prematurely based on one particular line of data. Process coding also directed the analysis to take into account multiple participants’ perspectives. *Initial codes* were created for each line of 10 of the 28 transcripts. Then, we identified the most significant and most frequent codes (called *focused codes*, which are similar to themes based on grounded theory methodology) and used them to sort codes with similar meanings into groups. Symbolic interactionism enhanced our analysis because it provided guidance for asking ourselves questions during analysis of the data about women’s interactions with the characters and with themselves as they experienced the web-based app. This helped us to identify what aspects participants found meaningful. Then, we developed the focused codes into categories. The qualitative software Atlas.ti (qualitative data analysis; Scientific Software Development GMbH) [46] facilitated the analytic process by serving as a platform to organize data so that it could be easily shared between researchers. Excel charts facilitated our use of a constant comparison of data with data and codes with codes. We were limited to only one interview per participant, so we could not perform theoretical sampling. However, the techniques of grounded theory methodology allowed us to scrutinize every line of the very rich data we had collected to perform a systematic and rigorous analysis. Memo writing deepened the analysis, facilitated discussions about patterns in the data, enhanced researcher reflexivity, and led to checking potential biases throughout the analysis. From this, we were able to develop a robust description of categories with nuanced properties. Diagrams were instrumental in illustrating processes and identifying relationships between and within categories.

## Results

### Participant Details

Demographics and levels of depression and anxiety symptoms at screening for our sample of 28 Latina participants are reported in Table 1 [28]. More than half of our sample reported watching story-based dramas or comedies on television once or more each week and watching videos, movies, or story-based shows on the internet at least weekly, using a smartphone, tablet, or computer.

**Table 1:** Sample demographics (N=28).

Characteristic			Value, n (%)	
**Highest education level**				
	Some high school (but not all)			3 (11)
	Graduated from high school or earned a general educational development certificate			7 (25)
	Some technical, trade, or vocational school after high school			3 (11)
	Attended at least one course in college			8 (29)
	Graduated with an associate degree			1 (4)
	Graduated with a bachelor’s degree			3 (11)
	Graduated with a master’s degree			2 (7)
	Chose not to answer the question			1 (4)
**Finances**				
	**Ability to meet weekly financial needs**			
		Not difficult		3 (11)
		Somewhat difficult		6 (21)
		Very difficult		19 (68)
		Chose not to answer		0 (0)
**Mental health symptoms**				
	**Depression and anxiety**			
		Depression: PHQ-9^a^ score ≥10		3 (11)
		Anxiety: GAD-7^b^ score ≥10		4 (14)
		Depression and anxiety scores each ≥10		21 (75)

^a^PHQ-9: Patient Health Questionnaire 9-item.

^b^GAD-7: Generalized Anxiety Disorder scale 7-item.

### Moving From Passive to Active Participant in a Transmedia Storytelling Web-Based App Intervention

**Figure 1:**
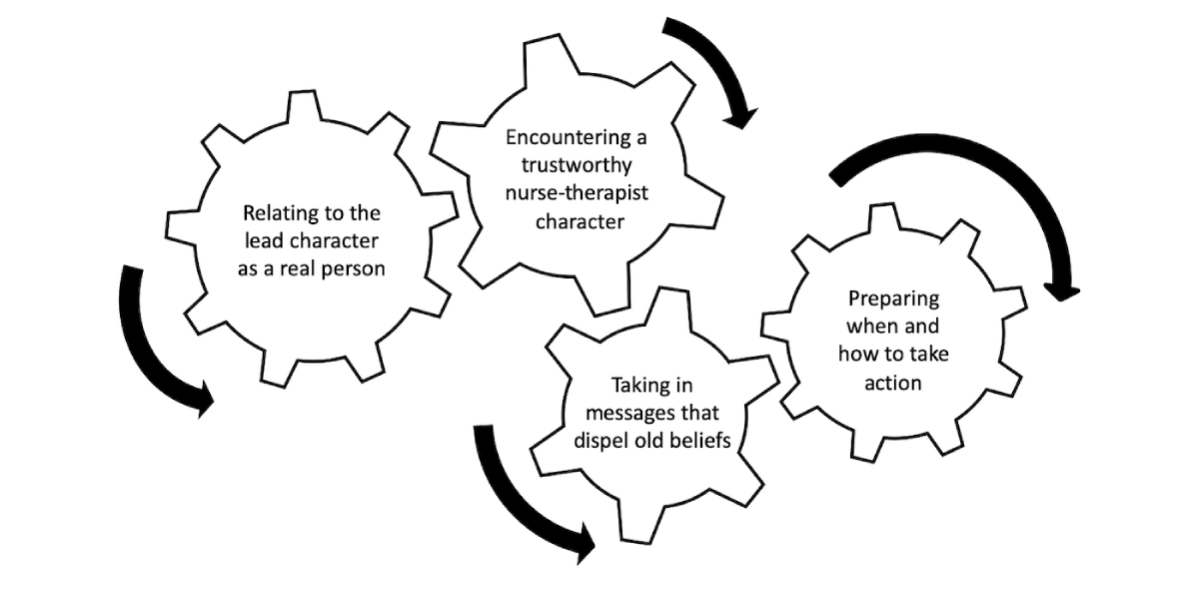
Depiction of the process of moving from a passive viewer to an active participant of the transmedia storytelling web-based app intervention.

The Latina women in our sample engaged in a multi-phase process that we conceptualized as *moving from passive viewer to active participant of a transmedia storytelling web-based app intervention*. This process is depicted using the metaphor of interlocking gears. The process begins with the movement of the first gear, which, in this depiction, is the symbolic connection to the lead character in the web-based app. Our sample of symptomatic Latina women found Catalina to be highly relatable (Figure 1). The analysis and description of this conceptual category was previously published [29]. We called it *relating to the lead character (Catalina) as a real person*. The turning of the first gear causes the next gear to turn, which then engages the next gear until all the gears are turning.

This analysis picks up after the first gear is in motion. In other words, having found Catalina to be relatable, the Latina women in our sample moved on to an active engagement with the second character, Veronica, a nurse therapist. This analysis provides a nuanced description of the second, third, and fourth phases of the process that Latina women went through upon meeting Veronica in the media of the web-based app. These 3 phases are depicted as interlocking gears and are representative of 3 new categories. These 3 categories give clarity to the process of moving from passive viewer to active participant. Each new category has 2 or 3 properties. The categories are as follows: *encountering a trustworthy nurse-therapist character*, *taking in messages that dispel old beliefs*, and *preparing when and how to take action*.

#### Encountering a Trustworthy Nurse-Therapist Character

**Figure 2:**
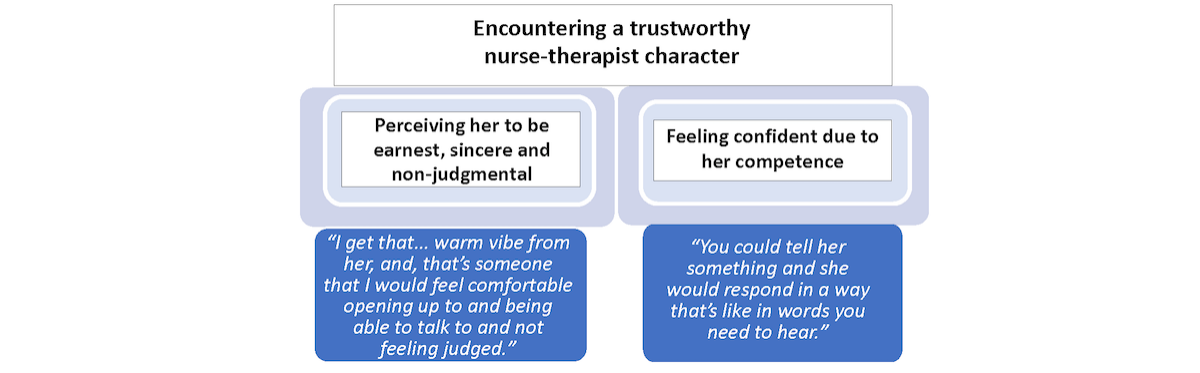
Properties of the category of encountering a trustworthy nurse-therapist character.

All participants clicked the links on the web-based app that extended the story from a focus on Catalina to bonus videos wherein Veronica, the nurse-therapist character, spoke directly to the audience. Our participants spoke at length about their experience of Veronica in interviews. They recognized Veronica as a trustworthy nurse who was also a capable therapist. As participants described their perceptions and reactions to Veronica, it was the element of trust that mattered most to them. This category had 2 properties: (1) perceiving her to be earnest, sincere, and nonjudgmental and (2) feeling confident due to her competence (Figure 2).

#### Perceiving Her to be Earnest, Sincere, and Nonjudgmental

Sincerity was perceived as a crucial aspect of Veronica’s trustworthiness. One participant described her as “very genuine” and indicated that Veronica’s choice of words and style of speaking conveyed an honest intention to help and a deep sense of caring. This made the media interaction feel enjoyable, comfortable, and safe. She stated:

She [Veronica] felt very eager to help. So, her tone of voice was soft but very straightforward but also like there was some love in it. So, I really liked hearing from her. I felt safe.

Participants did not focus on Veronica as a fictional character. Rather, they described how earnest she was, which impressed them. One woman said, “regardless if she really was a nurse, she seemed very sincere.” Participants explained that they felt certain that Veronica would *understand* instead of judging them, that they trusted her, and perceived her to be the type of therapist who would be *really open to listening*. In the context of their recent emotional struggles, the participants valued her lack of judgment, implying that it was not just important but necessary for them to feel comfortable.

Women described Veronica’s manner, how she looked, and how they felt in response to her demeanor. For example, one woman explained that Veronica seemed “warm and approachable,” that “her demeanor was very welcoming and very caring,” and although this participant said she typically would have preferred a male counselor, Veronica “felt like she was like someone that I could go and just share how I’m feeling” as she seemed to “be neutral and impartial.”

Putting the emphasis on the effect Veronica had on her, another participant said, “she seems to calm my nerves.” After recounting how she perceived warmth from Veronica, another woman emphasized her personal expectations and then projected feelings she anticipated having in the presence of Veronica:

I get, that - for me - warm vibe from her [Veronica], and, that’s someone that I would feel comfortable opening up to and being able to talk to and not feeling judged.

#### Feeling Confident Due to Her Competence

The second property of trustworthiness in the nurse character was her competence, which inspired confidence. Participants believed that Veronica embodied different facets of competence, which made them feel confident about her. One woman said, “and, that's why I could feel comfortable—the way she sounded confident and [like] someone you can talk to, feel comfortable with.” Another said she was “very empathetic, very professional.”

Many were impressed by her expertise as a nurse therapist saying, “she’s very knowledgeable” and “it looked like she knows what she’s doing and talking about.” Another summed up Veronica’s proficiency by declaring firmly, “she’s great at what she does.” Participants commented on her presence as a skillful therapist and regarded her as a master:

Veronica seemed to be a person that has studied, I guess, human behavior in difficult situations. She seems like she is a person that is capable of helping a person in need and she seems to be trustworthy.

Participants also viewed her as a capable therapist who had a wide range of skills, able to help “no matter what” type of problem was presented. Her capability engendered confidence as one woman exclaimed, “I feel like she would give me a lot of good advice for my life, and how to deal with situations.” Several women expressed assurance that Veronica could handle any sticky or complex situation they might encounter. One woman stated:

. . ..because Veronica knows how to deal with problems like that, so I know she would be someone that could definitely help me over the problems, whatever I’m going through.

Others expressed themselves by describing a hypothetical scenario and then explaining how Veronica could tailor her skills to match the needs of such a person with such a problem at such a moment. One woman imagined confiding in Veronica and said “…you can tell you can trust her. You could tell her something and she would respond in a way that’s in words you need to hear.”

#### Taking in Relevant Messages That Dispel Old Beliefs

**Figure 3:**
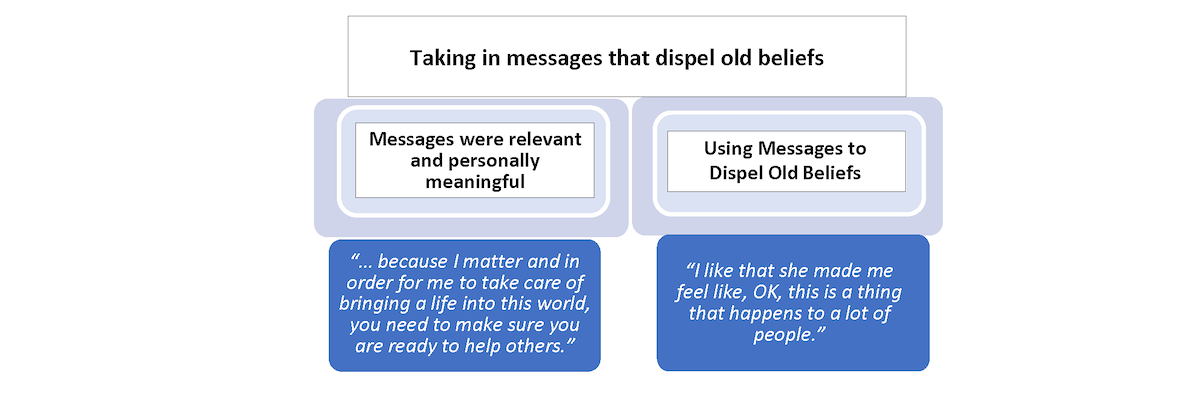
Properties of the category of taking in relevant messages that dispel old beliefs.

Our participants reported that the messages in the videos that involved Veronica were meaningful to them. How they described this content indicated that the messages were effective in bringing them new insight and deeper understanding of mental health. Thus, the second category is *taking in relevant messages that dispel old beliefs*. The 2 steps of this process are represented by 2 properties of this category: (1) taking in new messages because they were personally relevant and meaningful and (2) using these messages to dispel old myths and erroneous beliefs about mental health (Figure 3).

#### Messages Were Relevant and Personally Meaningful

When the interviewer asked general questions about participants’ thoughts related to seeking help for emotions in general, participants spontaneously answered in a way that integrated Veronica’s messages. They personalized the content of Veronica’s messages to fit their own situation. Remarkably, they remembered her messages even though the web-based app had not prompted them at any point to memorize or recite any content in the app. Furthermore, when asked how they felt about Veronica, they responded as if Veronica and her messages were so intertwined that they were the same thing. For example, when asked how she *felt* about Veronica, one participant did not answer by describing a feeling. Rather, she answered by citing one of Veronica’s messages that she found relevant in her life and related it to her own family, saying:

I liked the point that she [Veronica] brought up, I believe she had said, “First,” you know, “you have to make sure you’re okay with yourself, and you need to seek help if you want to,” you know, “have a better life and also help your family.”

The content delivered by Veronica in the web-based app seemed to be taken in and absorbed by the women. During the interviews, women transposed what Veronica said by putting it into the first person as if it was their own idea, never mentioning that they were using Veronica’s words. Sometimes during an interview, women shared something Veronica had said as if they were educating the interviewer. For example, one woman who was pregnant at the time of the interview gave a brief description of Veronica and then quickly went on to paraphrase one of Veronica’s messages saying, “...because I matter, and in order for me to take care of bringing a life into this world, you need to make sure you are ready to help others.” With conviction, this participant explained the reasons why seeking therapy would be crucial for her and her family. She shared her *own* view but never mentioned that Veronica had said very similar words in the video.

Another participant described how she “wished” she could talk with a person like Veronica when “problems in the home” occurred. She reiterated Veronica’s advice from the video and expanded upon it, bringing in how emotional struggles can take a toll on one’s body, which she said was why taking action felt specifically relevant to her. She said:

She gave really good advice. Like, getting therapy is good. It’s good to get help. It helps. You read, you take it out, you take it out of your body. It’s not good to be holding things in. It’s good to talk to somebody, you know? It helps. It makes things better. You feel it helps you more be less stressful, less nervous, you feel like a different person.

#### Using Messages to Dispel Old Beliefs

Through the process of taking in new messages, the women experienced a shift in thinking that spurred new insight. In the process, it dispelled old beliefs and erroneous myths about mental health and mental health care. Women said that Veronica clarified how common mental health challenges were and in doing so “put things into perspective.” They explained how Veronica helped them see their symptoms differently, which made them feel more comfortable and less odd or unusual. One woman said, “maybe it’s more normal than we think!” and then added, “I like that she [Veronica] made me feel like, OK, this is a thing that happens to a lot of people.” Her experience with Veronica dispelled a previous belief that she was the only one, as it did for many participants. Another woman with the same realization summed it up saying, “...we’re not alone. There’s a lot of other women out there that are going through the same thing.”

One woman dispelled an old myth that had prevented her from seeking help “until” she felt it was finally time. She shared her feeling that she had to wait until the right time, but she never seemed to know when the time was right. Referring to how, over time, she quietly endured emotional struggles that seemed to multiply, she said, “I guess I feel like we take it in, we take it in until we finally burst.” She then explained how Veronica’s messages helped change her perspective about the right time to seek help. She said:

I guess after seeing these videos and seeing the way the therapist talks, that’s what, made me feel comfortable to know that you don’t have to wait until you can’t take it anymore. You could get help before so that you won’t get to that point.

Participants described how Veronica’s messages brought awareness, saying that she “made me realize” things. In the context of the overall story line in the web-based app, another participant shared how she realized a key point about therapy. Previously, she thought that if she went to a therapy appointment, she would be doing all the talking. Now she realized that actually, “maybe it’s more of a combination where we’re both talking.” This and the other details that Veronica shared about getting mental health care were practical and helpful, as one woman said:

...she explained how it would help to get therapy, what the benefits are to talk to someone, how it would make you feel better and possibly change your life or even your lifestyle.

These details, coming from Veronica, dispelled the idea that getting help was not acceptable, and that, in fact, it could lead to a positive change in their personal lives.

#### Preparing When and How to Take Action

**Figure 4:**
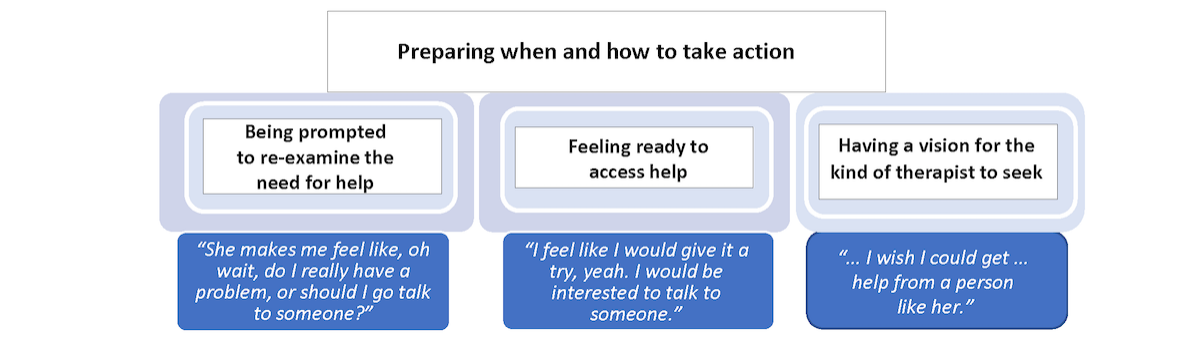
Properties of the category of preparing when and how to take action.

After the women decided that Veronica was trustworthy and engaged in the process of integrating messages they found to be relevant, they entered a part of the process that involved preparing when and how to take action. This category has 3 properties: (1) being prompted to re-examine the need for help, (2) feeling ready to access help, and (3) having a vision for the kind of therapist to seek (Figure 4).

#### Being Prompted to Re-examine the Need for Help

After finding Veronica to be trustworthy, participants found that the content of her messages helped them reflect to gain a more enlightened view of themselves. They felt prompted to apply what they learned from Veronica and to step back, take an honest look at their ability to cope with their emotional struggles, and examine it in the context of their life circumstances. One woman with 5 children, who never sought therapy before and always put her children’s needs before her own, was moved by Veronica’s message. This woman said, “She [Veronica] gives good advice, and I just feel everything she said is true.” She went on to discuss the possibility of finding a therapist saying, “I get to thinking, I am thinking about it... They [the videos] give you good information, like the counselor [Veronica], and it’s true, it’s good to talk to people.”

Another participant reflected on how she felt before engaging with the transmedia web-based app intervention saying, “I’ve never been, ‘OK, I’m going to go’.” However, she continued to say that now she found herself open in a way that was new for her. Admitting that, thanks to Veronica, she had re-evaluated her need for mental health care, she said, “She makes me feel like, ‘Oh wait, do I really have a problem?’, or ‘Should I go talk to someone?’” Her willingness to be open and inquisitive about her own need for help was key to moving forward in the help-seeking process.

#### Feeling Ready to Access Help

The second property of preparing to get help involved feeling ready. Some participants, who announced their unequivocal desire to seek help after engaging with the web-based app, attributed their decision to the character, Veronica. This included her presence overall and her insightful messages. One woman, struggling to cope with her life as a single mother said, “I’ve been having a lot of anxiety due to all my stress, just overall in my life.” She expressed her enthusiasm and enhanced readiness to seek therapy by saying:

I guess just seeing Veronica and the way that she is and how she brought up good points. So, it’s something that I would definitely be interested in doing.

After coming to terms with Veronica’s messages, another participant indicated that she was more prepared to take the risk of seeking help. She said, “I feel like I would give it a try, yeah. I would be interested to talk to someone.” Another woman gained hope and optimism that therapy had the potential to improve her life:

Actually, all the stuff that she said, it’s true. You shouldn’t feel alone. You should always get help, especially when you’re feeling down or depressed. If you get help, it's going to help you a lot, it's going to help you a lot. I don’t know yet, because I haven’t gone to get help, but I am pretty sure that if I could get help, I know it's going to help, and I know that's what I need.

#### Having a Vision for the Kind of Therapist to Seek

The third property of preparing to get help involved desiring to find a therapist like Veronica. Participants were so moved by Veronica that some not only wanted to seek professional help but also wanted to find a therapist who was like her. One woman, who revealed that Veronica changed her thinking about seeking mental health care, shared that feeling comfortable with a therapist was a priority. She felt that it would maximize her ability to open up and talk more freely. She stated:

I was thinking about, I wish I could get in contact or help from a person like her. It makes me feel kind of, you know, when you feel fine about someone, that you can talk?

Another participant expressed how the web-based app’s depiction of a therapist like Veronica actually gave her hope. It provided the impetus to take the initiative to seek help for herself. She stated:

I wanted to believe that there will be another therapist like her as well. You know, like, that understands you. So, it just made me feel comfortable to actually take that step.

Another woman who had thought about seeking help “plenty of times” but never followed through said, “I just worry so much, like, if they’re really going to understand me.” However, watching Veronica, she said, “If I had a therapist like her, I would not have a problem meeting with a therapist, honestly.” This helped generate the motivation to plan for her course of action to enter therapy. As a result, she planned to “talk to my doctor. He’ll recommend someone nearby my house.”

## Discussion

### Principal Findings

Our web-based app, *Catalina: Confronting My Emotions*, offered our Latina participants a culturally acceptable digital bridge that ultimately led them to resources for getting the needed mental health care. Although the first step of the process of engagement with our web-based app involved participants identifying with Catalina, it was Veronica, the fictional nurse-therapist character, who served as the catalyst through the remaining steps of the process that led participants to options for action*.* Our sample became actively engaged with the web-based app and transitioned from the point of view of the *observer* to that of the *experiencer*, moving from a passive position of watching to an active engagement, guided by Veronica. This process included imagining, thinking, reflecting, and acting. No doubt the process of engagement started when participants recognized themselves in the relatable lead character of the story line, Catalina [29]; this put the metaphorical gears of the dynamic process in motion (Figure 1). However, it was engagement with Veronica, the nurse-therapist character, that soon became the focus of their experience as they clicked to open transmedia bonus videos and other features. Through this, participants shifted from a focus on how Veronica helped Catalina in the story to personally experience how Veronica was actually helping them in real time. As such, Bandura’s dynamic of social modeling [26,47] was in motion first through Catalina and then through Veronica. By increasing engagement with the nurse-therapist character (Veronica), participants were able to apply her messages to themselves.

As Veronica’s messages mattered to them, they digested them. They came from someone they saw as a knowledgeable, competent health professional, who they wanted to listen to and engage with. This messenger, Veronica, also seemed to really care. She was a nurse and a therapist, but, importantly, she was also a Latina woman who understood them. Veronica’s messages prompted participants to replace previous assumptions with new perspectives on the healing potential of therapy and other mental health resources. As the old misconceptions about mental health were defused, the gears turned onward. The next phase of the dynamic process led participants to begin preparing themselves to take action. They were able to focus on when, how, and what they could do to make a change in their lives. The potency of Veronica’s messages added to the power of our web-based app to become a conduit to care for our sample, over a third of whom took action to use a resource, get information, or make an appointment within one week of engagement [28].

As participants already found the Catalina character to be highly relatable, the bar was set high for any characters who followed in the web-based app’s transmedia bonus videos. The results of our analysis showed that Veronica attracted and held the participants’ attention, deepening engagement and investment in the web-based app experience. There is no doubt that this is in part due to the talent of the Hollywood actress who portrayed Veronica (Yareli Arizmendi). Participants saw Veronica as welcoming not only because of how she looked or the sound of her voice but also because of the way she conveyed a sense of loving care to participants. From a film-directing point of view, Veronica was meant to be a knowledgeable character who was also compassionate [48]. Data from participants indicated that Arizmedi’s performance met the mark.

As we analyzed the transcripts from interviews with Latina women in this study, we found it impossible to tease apart participants’ views of the character (Veronica) from the content of the messages she delivered. Participants seemed to perceive them as being integrated as one and the same. Many women incorporated the actual language used by Veronica into their own self-expression. Women voiced her words as their own, which suggests that the things she communicated rang true to them, most likely because they were relevant to their lives. This underscores the importance of partnering with members of the target group to allow *their* concerns to be central to the design of a storytelling mental health web-based app [21,22,49]. It also indicates that casting a talented actress who could powerfully and poignantly embody a character was vitally important.

Our scripts were based on the priorities of Latina women who had struggled with depression that we had identified in previous research [30,31,36-38]. This included Latina women’s own past experiences with depression when alone or in therapy sessions with a therapist as well as lessons they learned and found valuable. As noted earlier, well before filming, the scripts were written by a Latinx script writer from Hollywood and revised with input from therapists. After filming, the first cut of the videos was vetted in theater testing by a sample of Latina women who fit the demographics of the target group [28]. This was done before our mixed methods study was implemented with symptomatic Latina women. The input from our participants for this analysis implies that the script indeed reflected what they needed to hear and that content was delivered in a way that they found easy to receive and absorb. Taking the messages from the script into account was not a passive act for them. Rather, the participants took an active role in the web-based app experience, considering its video content, as the story unfolded, clicking to watch each of the bonus videos and engaging in the interactive questions on screen as the story extended from a focus on Catalina to a focus on Veronica to a focus on them.

Participants became aware that distressing symptoms warranted professional help and their engagement with the web-based app expanded their vision of both the kind of help they wanted to find and where to get it. Interestingly, these are similar to the elements of mental health literacy that Jorn [13] described. It is possible that what helped facilitate this process was the compassion that the character, Veronica, expressed in her portrayal of a nurse therapist. Kemp et al [48] performed an analysis of compassionate mental health care delivered in digital form, such as through web-based apps. They defined compassion as having several elements that seem to pertain to the way that Veronica was perceived by Latina women in our sample. For example, through her gestures and words, Veronica conveyed awareness that many women have emotional struggles and need help. Participants saw Veronica as focused on helping by reducing suffering. Veronica also encouraged them to click the next link, engage with the interactive sequence, and think about what was holding them back. Participants could have chosen to stop the web-based app engagement at any point in time and just turn it off. However, they continued, likely because of their connection to Veronica and because engagement felt useful to their own journey [21]. Although simultaneously being knowledgeable and caring in a way that Latina women welcomed, Veronica also modeled mental health literacy; she shared information about depression and anxiety, options for treatment, and where to get help [13]. Women responded with high interest, trust, and, ultimately, action.

Engagement with this web-based app created a unique space for participants, one that helped optimize mental health contemplation and led to action. It provided our Latina participants with an opportunity to experience what it might be like to have Catalina’s therapist interact with them. In the interactive sequence of 5 short 2-minute videos [28], Veronica posed questions from the confidence ruler and the importance ruler based on motivational interviewing [41]. Participants could move through this sequence at their own pace, replay a video, or return to it later. This gave participants the choice to engage with Veronica through a back-and-forth experience of videos and questions. It included ample space for personal introspection, possibly contributing to their feeling that this might be what an actual therapeutic interaction felt like. Participants valued this experience, which further enhanced engagement; it is noteworthy that we had no attrition in this study [28].

Torous et al [14] have called for the creation of digital technologies that lead to increased access to care while fostering a therapeutic relationship. Our character-driven web-based app holds promise for moving in that direction. For example, although our purpose was to engage symptomatic Latina women and then connect them to sources of therapy and help, our results showed that engagement in our web-based app led to statistically significant drops in depression and anxiety scores, both 1 and 6 weeks after engagement [28]. It is possible that this was partially due to a kind of therapeutic alliance that women developed with Veronica. Although she was not providing live therapy to participants, she was interacting therapeutically with them to enhance their self-awareness, confidence, and sense of the importance of getting needed care. Veronica also normalized mental health symptoms and helped women feel less odd, thereby challenging the stigma associated with depression and anxiety. Overall, Veronica met the participants’ needs.

### Limitations and Next Steps

The findings of this analysis are not meant to be generalizable to all Latina women or to all Latina women with mental health symptoms. In addition, it is not generalizable to users of story-based apps, character-driven apps, or transmedia apps. Nonetheless, the knowledge generated here advances an understanding about important elements of mental health provider characters featured in storytelling web-based apps for underserved groups, specifically, English-speaking Latina women with depression and/or anxiety. Future research with a Spanish version of our web-based app is needed to test feasibility, acceptability, and efficacy with a sample of Spanish-speaking Latina women. In addition, a future randomized controlled trial with a much larger sample that is broader is needed to validate the study with evidence from symptomatic but untreated Latina women, both Spanish and English speakers, in comparison with an attention control. Potential extensions of this work include using a theory-driven approach to create additional character-driven webisodes or bonus videos that extend the story while supporting participants who are engaged in needed therapy (actual or virtual). Other interactive modules that are character-led and therapeutic could be created and tested in English and Spanish. Further exploration, development, and experimentation hold promise for gaining needed insight into the most potent uses of story-based features in mental health web-based apps. Finally, testing the web-based app with women of other ethnic groups or translation of the web-based app into languages other than English and Spanish would provide valuable insight related to helping other populations. Diverse, multidisciplinary teams will be crucial as we advance the science of web-based apps for health that use transmedia storytelling.

### Conclusions

As researchers and developers continue to produce mental health apps, research and testing are crucial to identify the areas where we need to focus our efforts to provide effective web-based or app-related tools [23]. Our web-based app was both a useful conduit for mental health resources and an experience that was dynamically experienced by symptomatic Latina participants. Our positive results to date are likely due to many things including the theory-informed [26,45] process of development that we undertook and the multi-layered approach of drawing upon input from Latinx collaborators at every step. This analysis of qualitative data collected after use of the web-based app by the target group about their experience is crucial for informing the next step of app development. Although a theory-driven, collaborative, data-based approach is time consuming, it is key to creating web-based apps that are desirable and acceptable to a target group such as symptomatic Latina women and, therefore, more likely to be successful in meeting their needs.
